# Type 2 diabetes and inflammatory bowel disease: a bidirectional two-sample Mendelian randomization study

**DOI:** 10.1038/s41598-024-55869-x

**Published:** 2024-03-01

**Authors:** Guangyi Xu, Yanhong Xu, Taohua Zheng, Ting Liu

**Affiliations:** 1 School of Nursing, Qingdao University, Qingdao, 266071 China; 2 Department of Gastroenterology, The Affiliated Hospital of Qingdao University, Qingdao, 266003 China; 3 Cardiovascular Surgery Intensive Care Unit, The Affiliated Hospital of Qingdao University, Qingdao, 266003 China

**Keywords:** Type 2 diabetes mellitus, Inflammatory bowel disease, Mendelian randomization, Clinical genetics, Genetic association study, Inflammatory bowel disease, Risk factors

## Abstract

To investigate the association between T2DM and IBD by bidirectional two-sample Mendelian randomization (MR) to clarify the casual relationship. Independent genetic variants for T2DM and IBD were selected as instruments from published genome-wide association studies (GWAS), mainly in European ancestry. Instrumental variables (IVs) associated with T2DM and IBD were extracted separately from the largest GWAS meta-analysis. MR analyses included inverse variance weighting, weighted median estimator, MR Egger regression, and sensitivity analyses with Steiger filtering and MR PRESSO. In the data samples for Ulcerative colitis (UC) (6968 cases, 20,464 controls) and Crohn's disease (CD) (5956 cases, 14,927 controls), there was a negative causal relationship between T2DM and UC [IVW, OR/95%CI: 0.882/(0.826,0.942), *p* < 0.001]. However, the causal relationships between T2DM and CD, UC and T2DM, CD and T2DM were not significant, and the p value measured by the IVW method was ≥ 0.05. All SNPs showed no significant horizontal pleiotropy (*p* > 0.05). The results of the bidirectional MR Study suggest that T2DM has a negative causal effect on UC, which provides implications for clinical treatment decisions in IBD patients with T2DM. The findings do not support a causal relationship between T2DM and CD, UC and T2DM, or CD and T2DM, and the impact of IBD on T2DM needs further investigation.

## Introduction

Inflammatory bowel disease (IBD) is a type of chronic intestinal inflammation mediated by abnormal immunity. Ulcerative colitis (UC) and Crohn's disease (CD) are the two main forms of IBD, which currently affect about 0.3% of the world's population, or more than 20 million people^1^. The incidence of IBD increased dramatically in the Western world during the twentieth century, but its incidence had leveled off by the twenty-first century^2^. The etiology of IBD is still unclear. Studies have shown that environmental factors play an important role in its occurrence and development. For example, the incidence of UC and CD is relatively low in Asia compared with North America and Europe. The prevalence of UC among European South Asian immigrants was similar to that among Europeans, whereas the prevalence of CD among European South Asian immigrants was lower than that among Europeans^3,4^. In addition, IBD is closely related to smoking, diet, oral contraceptives, and vaccination^5,6^. Similar with other chronic inflammatory diseases, IBD usually develops early in life and is accompanied by a host of sequelae and comorbidities, including rheumatic diseases, iron-deficiency anemia, and cancer, and is therefore a major contributor to the global burden of disease^7^. In addition, IBD is a systematic inflammatory state and is associated with significant comorbidities. Research showed that type 2 diabetes mellitus (T2DM) is similar with IBD in light of the risk factors which include genes, gut bacteria and lifestyle^8^. Research established a correlation between T2DM and increased severity of IBD. Specifically, IBD patients with coexisting T2DM had a higher risk of infection, increased utilization of medical resources, and decreased quality of life than those without T2DM, while currently there is no effective immunosuppressive therapy for IBD patients^8^.

Studies have shown that there is a clear clinical association between T2DM and IBD^8,9^. Research found that genetic susceptibility to T2DM can raise the risk of gastrointestinal diseases in patients^10^. Diabetes may negatively affect the course of IBD by adding the risk of hospitalization and infection, but does not increase IBD-related complications and mortality^11^. Similarly, IBD can also increase the risk of diabetes^12,13^. However, these studies only demonstrate some correlation between T2DM and IBD, and the causal relationship between the two diseases remains unclear. Given that T2DM is a relatively well controlled disease by using hypoglycemic agents, exploring the causal relationship between T2DM and IBD may shed light on the treatment for IBD.

Mendelian randomization (MR) is the use of genetic variation in nonexperimental data to estimate causality between an exposure and an outcome^14^. MR can examine the potential causal relationships from exposure to outcome using instrumental variables (IVs)^15^. The results of current MR analysis mainly rely on genome-wide association studies (GWAS) databases, usually referring to single nucleotide polymorphisms (SNPs), which are used as IVs^15^. Compared with traditional methods, MR analysis can minimize the effect of confounding factors on causal estimates because genetic variants are randomly assigned at conception, and genetic variants from parents remain unchanged after birth^16^. Recently, two-sample MR analysis has been applied to investigate relationship between IBD and many diseases (e.g., gut microbial genera, atopic dermatitis, depression, fatty acids, neurodegenerative diseases)^15,17–20^. However, previous epidemiological studies have not focused on the causal relationship between T2DM and IBD phenotypes. Bidirectional two-sample MR may help to reveal the complex causal relationship between the two diseases. Therefore, this study aimed to evaluate and analyze the causal effect between T2DM and IBD by performing a bidirectional two-sample MR analysis using pooled data from large-scale open access GWAS. The findings are expected to provide implications for taking measures to reduce the severity of IBD for patients with occurrent T2DM.

## Materials and methods

### Study design

**Figure 1:**
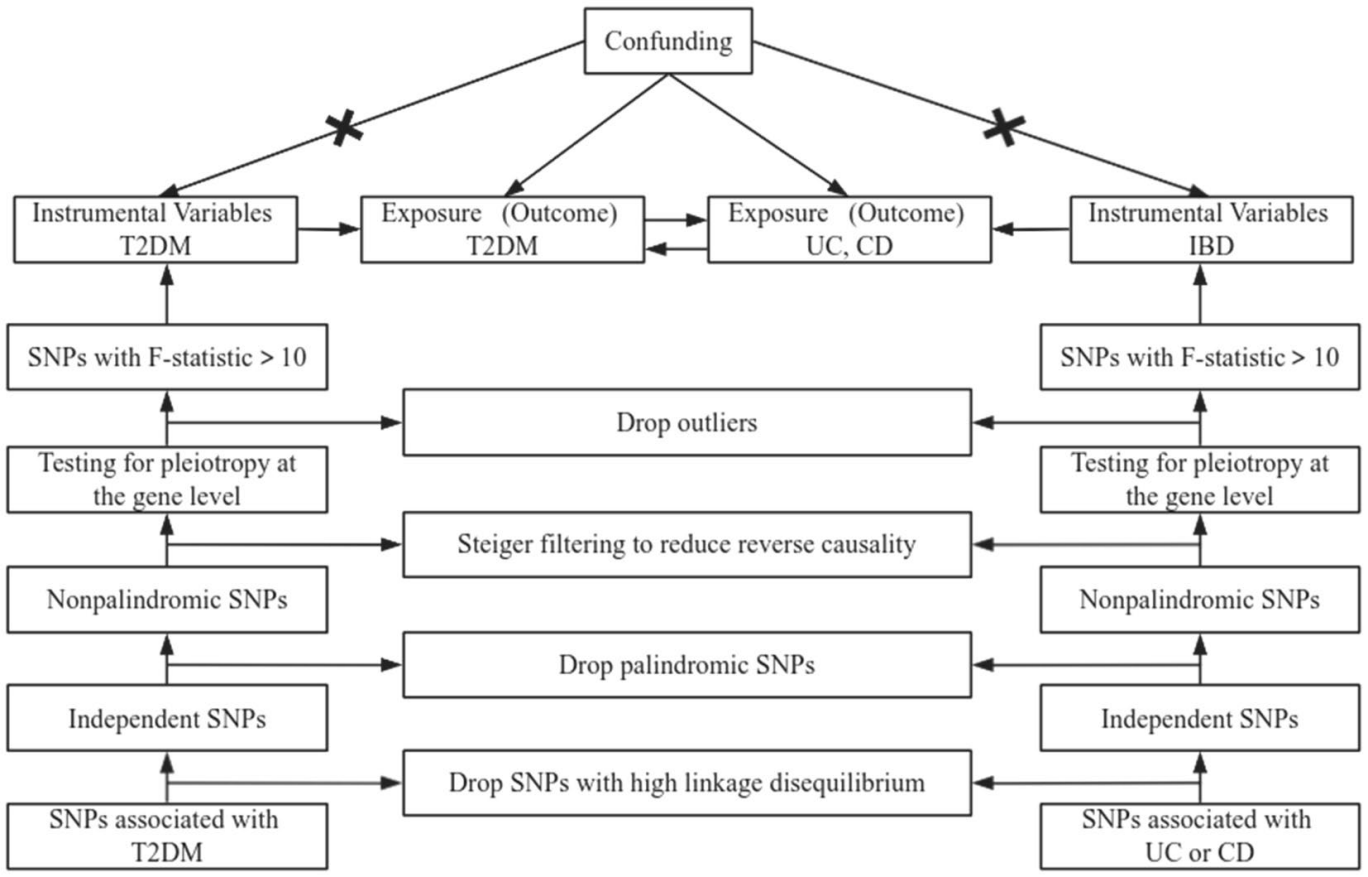
Overview of the present study design and results.

We investigated the association of T2DM with UC and CD using a bidirectional two-sample MR approach. According to the rationale and core assumptions of MR^21^, three hypotheses were followed in this study: (1) The genetic IVs must be strongly associated with the exposure; (2) SNPs were not associated with any confounding factors of the risk-outcome association; and (3) SNPs did not affect the results through any pathway other than exposure of interest. Figure 1 details a schematic representation of the design of this study. All data used in the study were from publicly available GWAS summary statistics. Ethical approval was obtained in all original studies. Therefore, no additional ethical approval or informed consent was required for this study.

### Data sources and instruments

#### T2DM

Summary data on the association of genetic variants with doctor-diagnosed T2DM were obtained from a recent GWAS meta-analysis of 5,053,015 SNPs in 62,892 patients with T2DM and 596,424 controls of European ancestry^22^. The study consisted of three contributing studies, including the Genetic Epidemiology Research on Aging (GERA), the Diabetes Genetics Replication and Meta-analysis (DIAGRAM), and the full cohort release from UK Biobank (UKB).

#### UC and CD

SNPs associated with IBD including UC and CD were obtained from the International Inflammatory Bowel Disease Genetic Consortium (IIBDGC) participants. The IBD GWAS statistics provided data on IBD overall (12,882 cases, 21,770 controls) as well as on CD (5,956 cases, 14,927 controls) and UC (6 968 cases, 20,464 controls)^23^. GWAS data were provided by IEU OpenGWAS database. This database is now available as resources for a wide range of analyses, such as MR Analysis^24^.

#### Selection of instrumental variables

We rigorously performed a series of quality control techniques to screen eligible genetic tools. First, the genome-wide significance level was defined as *p* < 5 × 10^–8^ to satisfy the correlation assumption so that the instrumental variables are closely related to the outcome. Second, to rule out variants in strong linkage disequilibrium (LD) and ensure the independence of each SNP, we used standard parameter SNPs (*r*^2^ < 0.01, window size = 1000 kb). SNPs in pairs with LD *r*^2^ values greater than the specified threshold (*r*^2^ > 0.01) and smallest p-values were retained. SNPs with minor allele frequency (MAF < 0.010) were eliminated. Third, SNPs (*p* < 5 × 10^–6^) associated with IBD were excluded by screening the GWAS catalog. Finally, ambiguous SNPs with discordant alleles (e.g., A/G vs. A/C) and palintic SNPs (e.g., A/T or G/C) were excluded from the process of reconciling exposure and outcome data sets.

### Statistical analysis

Prior to MR analysis, we calculated the F-statistic (F = [beta/SE]^2^) for T2DM IVs to quantify the strength of the instrument. The calculated results F-statistic > 10 indicate the absence of weak IVs bias^25^. We used inverse variance weighting (IVW) as the main analysis method for MR analysis. When the pleiotropic effect of IVs was not present and the sample size was large enough, the IVW estimates were consistent, valid, and close to the true value^26^. It has the most efficient instrumental variable analysis when all selected SNPs are valid IVs. The MR Egger intercept term was used to assess horizontal pleiotropy, where deviation from zero indicates directional pleiotropy. In addition, the slope of the MR Egger regression shows valid MR estimates when horizontal pleiotropy is present^27,28^. Complementary weighted median methods were used, which can show valid MR estimates by assuming that at least 50% of IVs are valid and ordering each IV's MR estimate as the inverse of its variance^29^. Thus, the weighted median estimator can provide reliable estimates. In addition, we performed several sensitivity analyses to examine and correct for the presence of pleiotropy in causal estimates. We performed the MR-PRESSO global test to assess horizontal pleiotropy and the presence of missing abnormal variants^30^. Cochran's Q was calculated to examine the heterogeneity of individual causal effects, with p-value < 0.05 indicating the presence of pleiotropy. Finally, we also performed an omission analysis to assess the impact of individual SNP_S_ on MR estimates. All analyses were conducted with R (version 4.2.2), R-based package ‘TwoSampleMR’ and ‘MR-PRESSO’.

## Results

### Instrumental variable statistical results

Through the above series of screening processes, 114 SNPs associated with T2DM and UC, 110 SNPs associated with T2DM and CD, 26 SNPs associated with UC and T2DM, and 37 SNPs associated with CD and T2DM were screened as IVs for UC and CD, respectively. The *F* statistics of IVs ranged from 29.942 to 1578.256, indicating a small likelihood of weak IVs bias. Details regarding all IVs are provided in Supplementary Tables S1, S2, S3 and S4.

### Primary MR analysis

**Figure 2:**
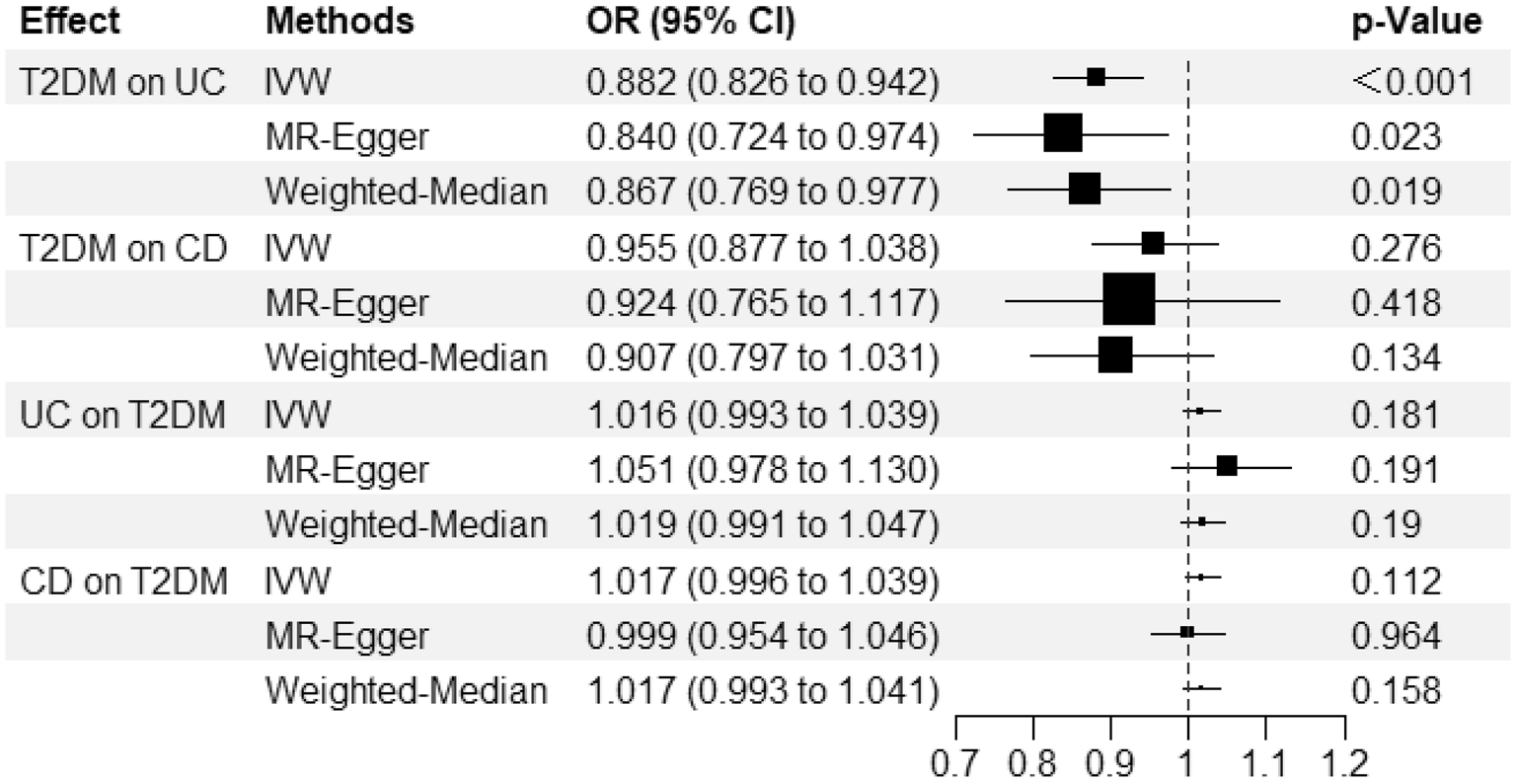
The forest plot about the causal associations between T2DM and IBD. *T2DM* type 2 diabetes mellitus, *UC* ulcerative colitis, *CD* Crohn's disease, *No. SNP* number of SNPs included in the analysis, *OR* odds ratio, *CI* confidence intervals, *IVW* inverse variance weighted.

MR-PRESSO method was used to perform the horizontal multiple effects test, and the outliers (*p* < 0.001) were removed for MR analysis. As shown in Fig. 2, there was a negative causal relationship between T2DM and UC[IVW,OR/95%CI: 0.882/(0.826,0.942), *p* < 0.001]. However, the causal relationships between T2DM and CD, UC and T2DM, CD and T2DM were not significant, and the p value measured by the IVW method was ≥ 0.05. According to the IVW approach, each standard deviation (SD) increase in genetically measured T2DM level was associated with a 12.5% reduction in UC risk.

### Supplementary and sensitivity analysis

In addition to the main IVW analysis methods, MR-Egger and weighted median estimation methods were used to verify the accuracy of the results. These supplementary analyses were applied to confirm the negative causal relationship between T2DM and UC (*p* = 0.023, 0.019, respectively). For each SD increase in T2DM measured by MR-Egger and weighted median estimation methods, the risk of UC decreased by 17.4% and 14.3%, respectively. In the mean time, the differences between T2DM and CD (*p* = 0.418, 0.134, respectively), UC and T2DM (*p* = 0.191, 0.190, respectively), CD and T2DM (*p* = 0.964, 0.158, respectively) were not found significant by these methods, as shown in Fig. 2.

Heterogeneity was measured by Cochran's Q statistic. As shown in Table 1, the results of the heterogeneity analysis indicated that significant statistical heterogeneity was detected among the genetic instrumental variables in terms of the effect of T2MD on CD (IVW, *p* < 0.001) and UC on T2MD (IVW, *p* = 0.042). Therefore, the multiplicative random effects IVW model was applied in these associations to calculate causal effects. In addition, no significant statistical heterogeneity was found between genetic instrumental variables in the effect of T2MD on UC (IVW, *p* = 0.153) and CD on T2MD (IVW, *p* = 7.381 × 10^–7^). Therefore, the fixed effects IVW model was used for the initial MR analysis.

**Table 1:** Results of bidirectional MR analysis of T2DM with UC and CD.

Effect	Methods	No. SNP	Q-value (*p*-value)	Pleiotropy-test (*p*-value)	MR-PRESSO RSSobs (*p*-value)
T2DM on UC	IVW	114	128.371 (0.153)	0.004 (0.473)	221.126 (0.263)
T2DM on CD	IVW	110	170.025 (< 0.001)	0.003 (0.712)	196.280 (0.946)
UC on T2DM	IVW	26	38.429 (0.042)	−0.006 (0.340)	41.386 (0.047)
CD on T2DM	IVW	37	92.448 (7.381 × 10^–7^)	0.004 (0.395)	101.371 (0.499)

*T2DM* type 2 diabetes mellitus, *UC* ulcerative colitis, *CD* Crohn's disease, *No. SNP* number of SNPs included in the analysis, *Q-value* Cochran’s Q statistic, *IVW* inverse variance weighted.

Furthermore, horizontal pleiotropy effects were tested to determine whether T2DM-related genetic tool variants could cause IBD through other potential pathways. As displayed in Table 1, no significant horizontal pleiotropy was found in the MR analyses (all *p* values ≥ 0.05), suggesting that this MR study are virtually unlikely to be affected by potential confounding pathways and thus the results are robust and reliable.

**Figure 3:**
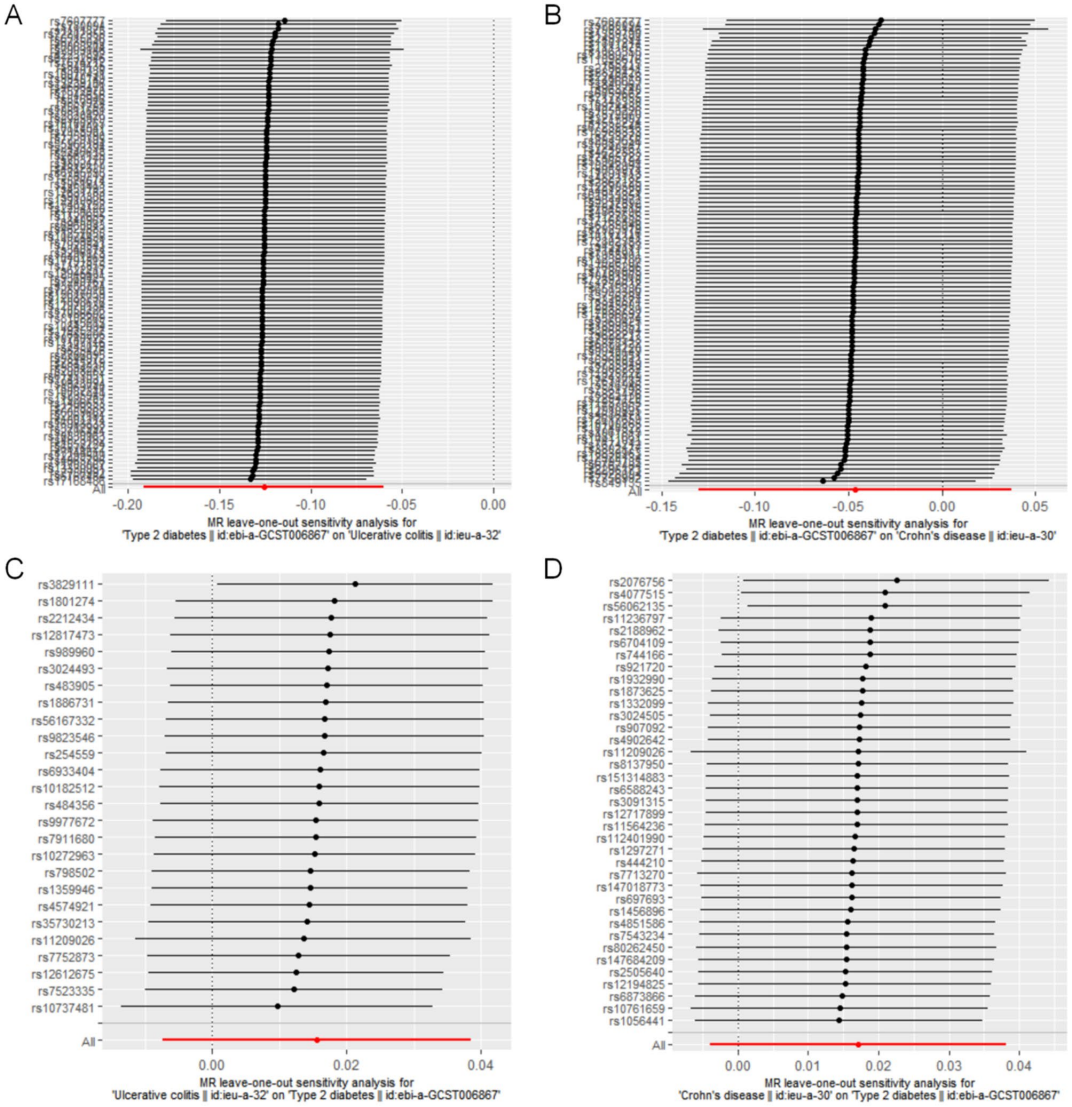
Leave-one-out analysis of the bidirectional effects of type 2 diabetes mellitus (T2DM) with ulcerative colitis (UC) and Crohn's disease (CD). (**A**) Analysis of T2DM on UC; (**B**) Analysis of T2DM on CD; (**C**) Analysis of UC on 2DM; (**D**) Analysis of CD on 2DM.

Leave-one-out analyses were performed to assess the effect of individual SNP_S_ on the final MR results. Figure 3 shows that the bidirectional residual causal effects of T2DM and CD and UC found in the omitted one analysis after sequentially omitting individual SNP_S_ were consistent with those found in the main MR study. This evinces that no single SNP played a significant role in the final results. The analyses further demonstrate that the MR study was robust, stable, and reliable.

**Figure 4:**
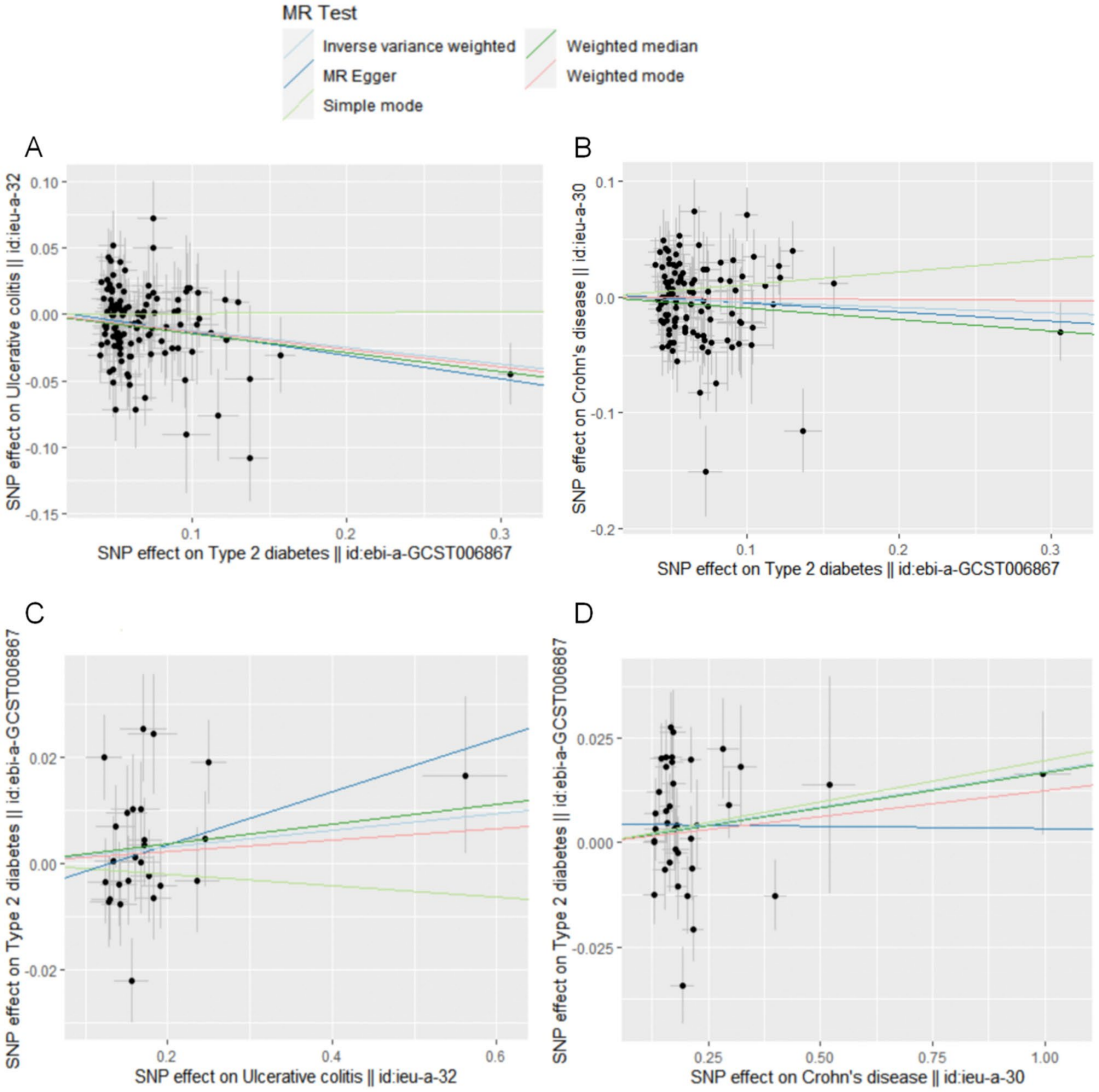
Scatter plots of the bidirectional effects of type 2 diabetes mellitus (T2DM) with ulcerative colitis (UC) and Crohn's disease (CD). (**A**) Analysis of T2DM and UC. The X-axis shows the single nucleotide polymorphism (SNP) effect and SE (standard error) for each selected SNP from the T2DM genome-wide Pooled Association Study (GWA) dataset. The Y-axis shows SNP effects and SE on UC from the UC genome-wide Pooled Association Study (GWA) dataset. (**B**) Analysis of T2DM and CD. The X-axis shows the SNP effect and SE for each selected SNP from the GWA dataset for T2DM. The Y-axis is SNP effect and SE on CD from the GWA dataset of CD. (**C**) Analysis of UC and T2DM. The X-axis shows the SNP effect and SE for each selected SNP from the GWA dataset from UC. The Y-axis is the SNP effect and SE on T2DM from the GWA dataset for T2DM. (**D**) Analysis of CD and T2DM. The X-axis shows the SNP effect and SE for each selected SNP from the GWA dataset of CD. The Y-axis is the SNP effect and SE on T2DM from the GWA dataset for T2DM.

Scatter plots were drawn to visualize the effect size of each MR method (Fig. 4) Forest plots were employed for the visualization of individual SNP estimates of the results (Fig. S1). Funnel plots were used to show the balance of the distribution of the effects of individual SNPs (Fig. S2). From these plots, it can be concluded that the effect and distribution of each SNP are balanced.

## Discussion

The present study used MR to analyze the bidirectional causal relationship between T2DM and IBD. The results showed that T2DM reduced the risk of UC, while the causal relationships between T2DM and CD, UC and T2DM, and CD and T2DM were not significant.

Chen et al.^10^ showed that for each unit increase in the risk of T2DM, there was a 0.93 unit decrease in the risk of UC. The present study confirmed that Type 2 diabetes could reduce the risk of UC, which may be related to the medications taken by patients with T2DM. For instance, Tseng^31^ followed 340,211 metformin users and 24,478 non-metformin users in remission of IBD for 5 years and found that metformin reduced the risk for recurrence of IBD in patients with T2DM. Deng et al. found that metformin reduced tight junction (TJ) protein expression in patients with T2DM and UC^32^. TJ proteins mainly exist in the junctional complex between epithelial cells and endothelial cells^33^. This structure connects adjacent cell membranes closely together and closes the epithelial cell space. Its function is to allow ions and small molecular soluble substances to pass through, and to prevent toxic macromolecules and microorganisms from passing through. The intestinal mucosal cells of ulcerative colitis highly express anti-inflammatory cytokines. When induced by anti-inflammatory cytokines, the expression of TJ protein will also increase, which promotes the permeability of intestinal mucosal cells to small molecule substances, leading to the occurrence of UC such as diarrhea^34^. Research demonstrated that IBD and T2DM share a common pathogenetic basis, which is affected by inflammatory processes, gut microbiota imbalance, and crosstalk between various signaling pathways^9^. Liu et al.^35^ found that metformin appeared to induce anti-inflammatory effects that improved symptoms of UC. In addition to the anti-inflammatory and antioxidant properties of metformin and enhancing intestinal barrier integrity in IBD cell and animal models, Wasuwit et al.^36^ revealed that metformin had the ability to restore the intestinal microbiota in mice with UC, thereby reducing intestinal inflammation. These evidences indicate that metformin can be used as an alternative therapy for IBD^36^, but its usage and dosage are still unclear. Therefore, future studies are warranted to further clarify its mechanism of action, usage and dosage, combination of drugs, and adverse reactions. Moreover, Pioglitazone shows potential benefits in treatment of IBD in preclinical studies^37^. Tseng^38^ evaluated the association between pioglitazone and major risk factors for IBD (psoriasis, arthropathy, dorsal paralysis, chronic obstructive pulmonary disease, and tobacco abuse) in 12,763 patients who had used pioglitazone and 12,763 patients with T2DM who had never used pioglitazone, and found no effect. However, rosiglitazone as a thiazolidinedione antidiabetic drug effective for the treatment of T2DM has been shown to be effective in the treatment of mild-to-moderate active UC^39^. Therefore, some drugs such as rosiglitazone could be prioritized owing to its dual effect during treatment for patients with T2DM and UC. Studies on the safety of some biologic agents and immunosuppressive therapies in patients with IBD and T2DM have been reassuring, yet newer and safer biologic agents that could reduce the risk of infection in such patients as first-line treatment need to be advanced^40^.

Despite that the present study established a causal relationship between T2DM and UC and provides implications for healthcare practice, the mechanism by which T2DM can reduce the incidence of UC rather than CD is still unclear. The study by Saadh et al.^41^ found that T2DM can increase the risk of CD. However, due to differences in cell biological factors, physical and chemical factors, genetic factors, and immune factors, the specific causal mechanism between T2DM and UC and CD can be further explored from these aspects through experimental methods in the future.

The results of this study suggest that the causal relationships between UC and T2DM, as well as CD and T2DM were not significant. This is consistent with the findings of Lai et al.^42^, who conducted a preliminary cohort analysis using the database of the Ministry of National Health in Taiwan while found no significant association between IBD and an increased risk of T2DM. Differences in study population and research methods may lead to disparity in these study results. Previous research have found that IBD can lead to an increased risk of developing diabetes in patients^12^. In a cohort study based on the Danish population, it was found that UC or CD increased the risk of T2DM in patients^43^. A study on a Korean population showed that the increased risk of diabetes in IBD patients was more prominent in younger age groups, and the risk of diabetes was higher in CD patients than UC patients^44^. García-Mateo et al.^45^ found that increased inflammatory behavior in CD predicted an increased risk of T2DM. However, these studies cannot confirm a causal relationship between UC and CD and T2DM due to the cross-sectional design nature. The present study found no causal relationship between UC and CD and T2DM by MR Analysis. This may be due to genetic differences in the study population. In addition to genetic variants, other factors can also increase the risk of IBD leading to T2DM. A cohort study of the IBD population suggests that the increased risk of developing T2DM in IBD patients may be associated with elevated disease severity^8^. Maconi et al.^46^ showed that the preferred treatment for active UC is corticosteroids, which may lead to glucose intolerance in patients, onset of diabetes, difficulty in controlling glucose levels, and complications in patients with diabetes. Therefore, the increased risk of T2DM in IBD patients may be ascribed to a variety of factors, and the conclusion regarding IBD causing the occurrence of T2DM cannot be reached. Although the current study did not find a causal relationship between IBD and T2DM, future research can start with the related factors of IBD and explore the association between IBD related factors and T2DM with large samples and multi-center methods.

This study has certain strengths. The study results confirmed a causal relationship between T2DM and UC. To the best of our knowledge, this was the first study to assess the bidirectional causal effect of T2DM on the development of IBD using a bidirectional two-sample MR approach. Firstly, the design of the study was based on three main instrumental variable assumptions and conformed to the checklist of the MR Survey^47^. MR approach is less susceptible to confounding, reverse causality, and non-differentially measured exposures than observational studies^48^. Therefore, the conclusions drawn in this study are reasonable and trustworthy. Second, both large-scale GWAS were obtained from European ancestry, which allowed avoiding bias of population stratification. In addition, a number of sensitivity analyses were performed to ensure the consistency of causal estimates and to confirm the robustness of the current findings.

Our study also has limitations. Pleiotropy is an important issue in MR studies. Our results do not appear to be affected by pleiotropy, as consistent results were obtained in sensitivity analyses, and few outliers were found using iterative IVW and MR-PRESSO methods. Second, this study only provides strong and reliable evidence for the effect of T2DM on IBD risk, whereas the bidirectional causal relationships between IBD and T2DM associated phenotypes such as fasting insulin, fasting glucose and haemoglobin levels were not analyzed due to limited access to these data. Future studies could address this limitation by applying for permission to using databases such as UK Biobank and Finnish data. In addition, because our study was limited to pooled data, the population was not categorized by sociodemographic factors, such as age, sex, or employment, when examining casual associations. Finally, our results were based on individuals of European ancestry while were not from the most recent GWAS database^49^, so the generalizability is limited. In the future, we can seek to utilize more comprehensive and updated GWAS databases to analyze the relationship between T2DM and IBD to validate the results.

## Conclusion

The results of the bidirectional MR Study suggest that T2DM has a negative causal effect on UC, which may be related to the use of metformin and pioglitazone, and thus can be considered as an alternative therapy for IBD patients. Given that the usage and dosage of metformin and pioglitazone are not clear in IBD patients, future studies are needed to further clarify its mechanism of action, usage and dosage, combination of drugs and adverse reactions. In addition, the causal relationships between T2DM and CD, UC and T2DM, and CD and T2DM were not significant. In future, the specific causal mechanism between T2DM and UC can be further explored by using experimental methods from the aspects of cell biological factors, physical and chemical factors, genetic factors, and immune factors.

### Supplementary Information

Supplementary Figures.

Supplementary Table S1.

Supplementary Table S2.

Supplementary Table S3.

Supplementary Table S4.

## Data Availability

The datasets supporting the conclusions of this article are available in the[repository name] repository. The GWAS summary statistics for T2DM is available on the website https://gwas.mrcieu.ac.uk/datasets/ebi-a-GCST006867/. The GWAS summarystatistics for IBD (including UC and CD) is available on the websites https://gwas.mrcieu.ac.uk/datasets/ieu-a-32/ and https://gwas.mrcieu.ac.uk/datasets/ieu-a-30/.The other data generated or analyzed during this study are available in this published article and its supplementary information files.

## Abbreviations

IBD: Inflammatory bowel disease
UC: Ulcerative colitis
CD: Crohn's disease
T2DM: Type 2 diabetes mellitus
MR: Mendelian randomization
IVs: Instrumental variables
GWAS: Genome-wide association studies
SNPs: Single nucleotide polymorphisms
GERA: Genetic Epidemiology Research on Aging
DIAGRAM: Diabetes Genetics Replication and Meta-analysis
UKB: UK Biobank
IIBDGC: International Inflammatory Bowel Disease Genetic Consortium
LD: Linkage disequilibrium
IVW: Inverse variance weighting
SD: Standard deviation
TJ: Tight junction
