# Supplementary material for: Type 2 diabetes and inflammatory bowel disease: a bidirectional two-sample Mendelian randomization study
Source: Sci Rep. 2024 Mar 1;14:5149. doi: 10.1038/s41598-024-55869-x (PMC10907708; doi:10.1038/s41598-024-55869-x)
Supplement: Supplementary file 1 — Supplementary Figures. [file 41598_2024_55869_MOESM1_ESM.docx]

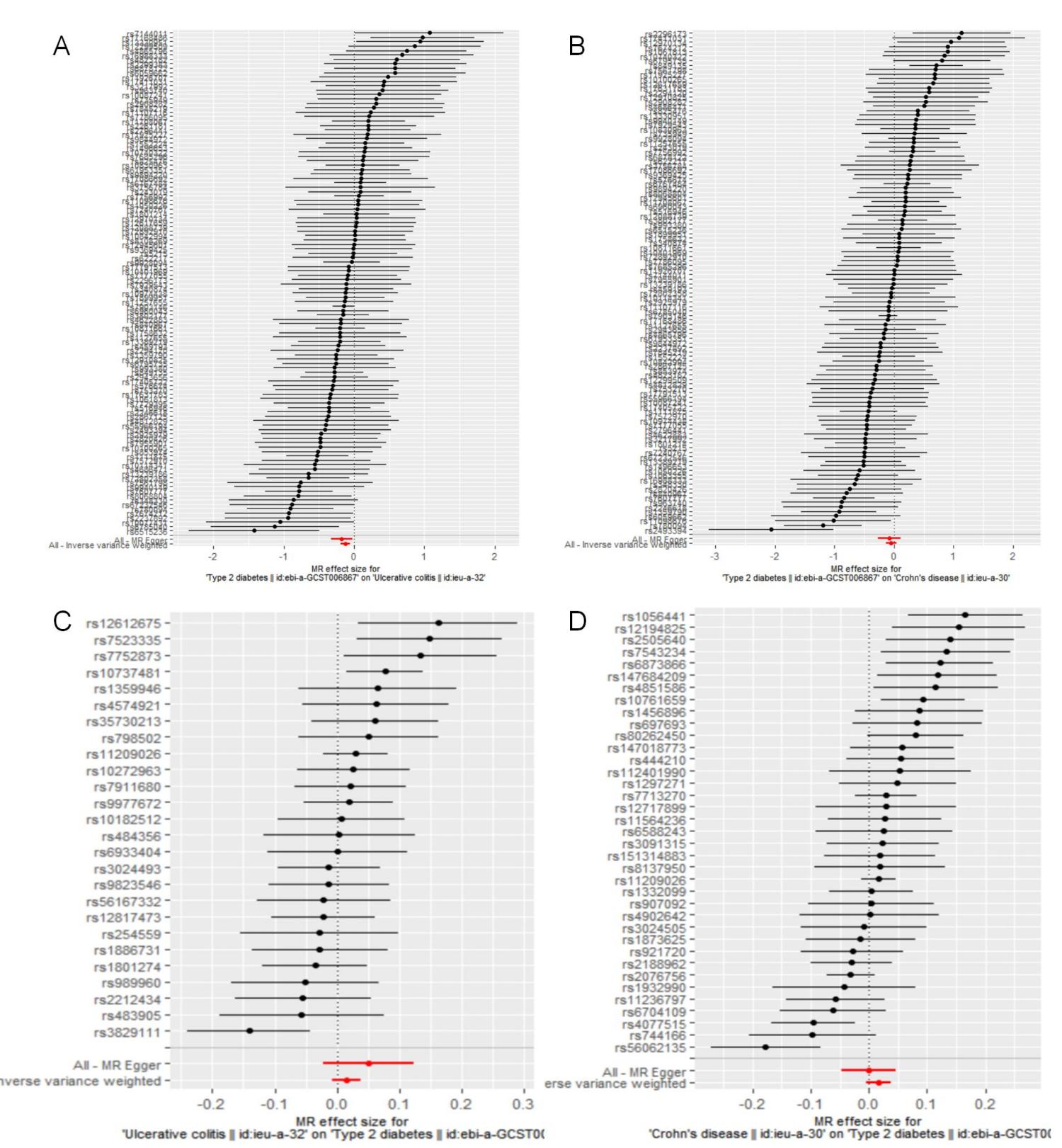


**Supplementary Figure S1.** Forest plots of two-way effects of type 2 diabetes mellitus (T2DM) with ulcerative colitis (UC) and Crohn's disease (CD). (**A**) Analysis of T2DM and UC; (**B**) Analysis of T2DM and CD; (**C**) Analysis of UC and T2DM; (**D**) Analysis of CD and T2DM.


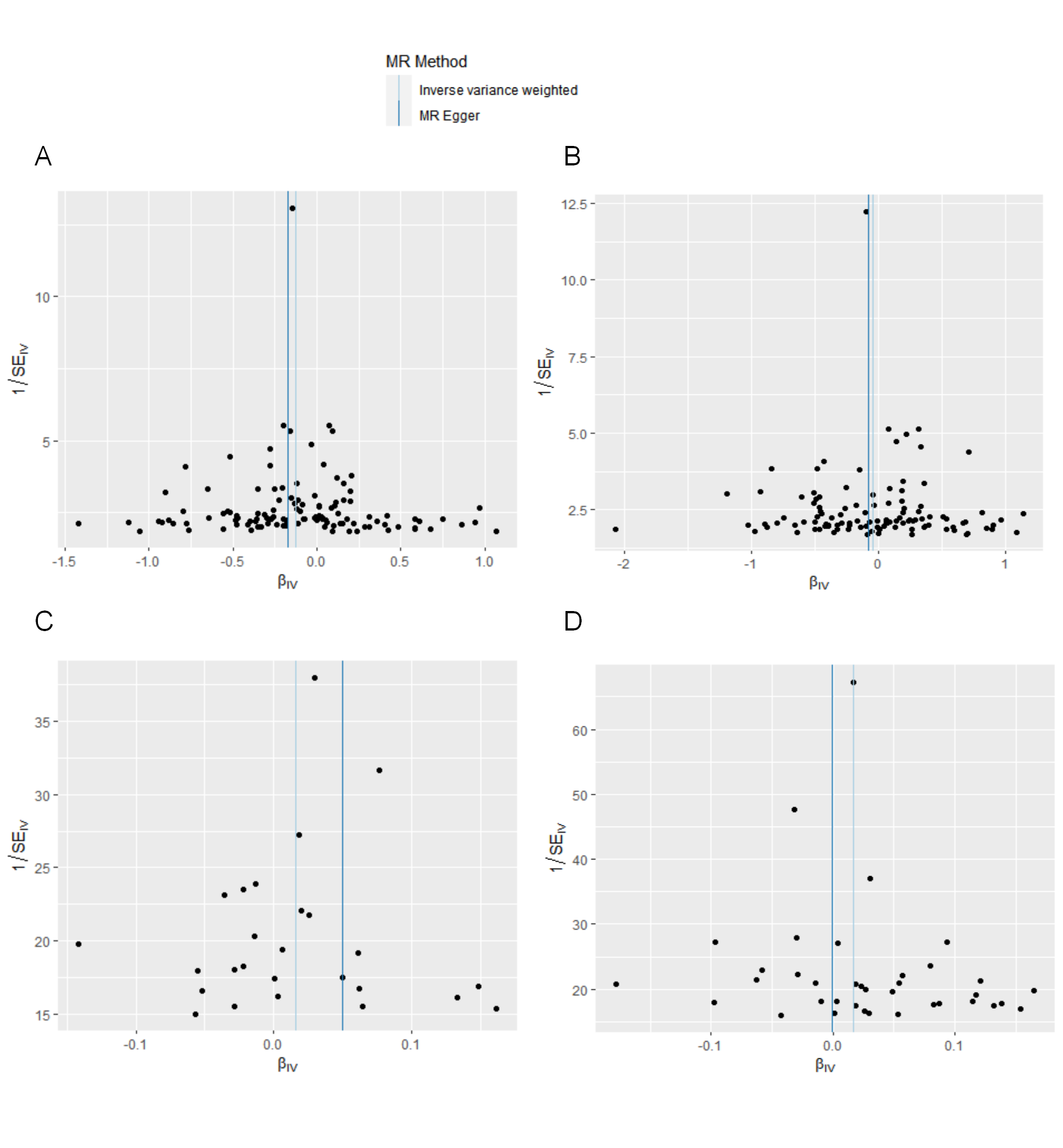


**Supplementary Figure S2.** Funnel plot of two-way effects of type 2 diabetes mellitus (T2DM) with ulcerative colitis (UC) and Crohn's disease (CD). (**A**) Analysis of T2DM and UC; (**B**) Analysis of T2DM and CD; (**C**) Analysis of UC and T2DM; (**D**) Analysis of CD and T2DM.
